# Assessment of a causal relationship between body mass index and atopic dermatitis

**DOI:** 10.1016/j.jaci.2020.04.050

**Published:** 2021-01

**Authors:** Ashley Budu-Aggrey, Sarah H. Watkins, Ben Brumpton, Mari Løset, Jess Tyrrell, Ellen H. Modalsli, Gunnhild Åberge Vie, Tom Palmer, Lars G. Fritsche, Jonas Bille Nielsen, Pål Richard Romundstad, George Davey Smith, Bjørn Olav Åsvold, Lavinia Paternoster, Sara J. Brown

**Affiliations:** a Medical Research Council Integrative Epidemiology Unit, University of Bristol, Bristol, United Kingdom; b Population Health Sciences, Bristol Medical School, University of Bristol, Bristol, United Kingdom; c K.G. Jebsen Center for Genetic Epidemiology, NTNU, Norwegian University of Science and Technology, Trondheim, Norway; d Department of Thoracic and Occupational Medicine, St. Olav's Hospital, Trondheim University Hospital, Trondheim, Norway; e Department of Dermatology, St. Olav's Hospital, Trondheim University Hospital, Trondheim, Norway; f Genetics of Complex Traits, Institute of Biomedical and Clinical Science, University of Exeter Medical School, Royal Devon and Exeter Hospital, Exeter, United Kingdom; g Department of Clinical and Molecular Medicine, NTNU, Norwegian University of Science and Technology, Trondheim, Norway; h Department of Public Health and Nursing, NTNU, Norwegian University of Science and Technology, Trondheim, Norway; i Center for Statistical Genetics, Department of Biostatistics, University of Michigan School of Public Health, Ann Arbor, Mich; j Department of Endocrinology, St Olav's Hospital, Trondheim University Hospital, Trondheim, Norway; k Skin Research Group, School of Medicine, University of Dundee, Dundee, United Kingdom; l Department of Dermatology, Ninewells Hospital and Medical School, Dundee, United Kingdom

To the Editor:

Atopic dermatitis (AD) is an itchy, inflammatory skin condition associated with multiple comorbidities. Observational epidemiology suggests an increased prevalence of obesity in patients with AD, but (1) whether there is a causal effect and (2) whether obesity leads to AD or *vice versa* remain unclear*.* Genetic predisposition to obesity has been shown to promote psoriasis,^1^ but dermatologic disorders can also lead to reduced participation in physical activity, resulting in weight gain. We aimed to investigate evidence of causality in the association of AD with elevated body mass index (BMI).

We meta-analyzed 33 published studies examining the association between obesity or elevated BMI and AD to summarize available observational data (see Fig E1 in this article’s Online Repository at www.jacionline.org). The odds ratio (OR) for AD in overweight individuals was 1.05 (95% CI = 0.94-1.19) in adults (n = 51,008) and 1.08 (95% CI = 1.00-1.16) in children (n = 506,202) (see Fig E2 in this article’s Online Repository at www.jacionline.org). For obese individuals, the OR for having AD was 1.19 (95% CI = 0.95-1.49) in adults (n = 1,400,679) and 1.20 (95% = CI 1.11-1.30) in children (n = 796,514) (see Fig E3 in this article’s Online Repository at www.jacionline.org); the methods and results are detailed in the Methods and Results sections of the Online Repository (at www.jacionline.org). We extended the observational analysis by using 2 large population-based studies from the United Kingdom and Norway^2^^,^^3^ (for details, see the Online Repository and Tables E1-E4). Among overweight individuals (BMI of 25-30 kg/m^2^), the OR of AD was 1.02 per each 1-kg/m^2^ increase in BMI (95% CI = 1.00-1.04; *P* = .07; 4,820 cases and 130,776 controls); a similar estimate was found among obese individuals (BMI >30 kg/m^2^) (ie, OR =1.02 [95% CI = 1.01-1.03; *P* = 3.3 × 10^–4^] in a sample of 2,741 cases and 73,907 controls) (see Fig E4 in this article’s Online Repository at www.jacionline.org).

Observational epidemiology has several limitations, including bias from confounding and reverse causation; this restricts its utility for causal inference. However, causality and the direction of effect can be investigated by mendelian randomization (MR). MR uses genetic variants as a proxy for the exposure (eg*,* BMI) to estimate the effect on an outcome (eg, AD). Genetic variants are randomly allocated at fertilization, therefore avoiding confounding; they are not affected by outcomes later in life, thus avoiding reverse causation. Genome-wide association studies (GWASs) have identified single-nucleotide polymorphisms (SNPs) associated with BMI (≤941 loci^4^^,^^5^) and AD (24 loci in European populations^6^). These SNPs can be combined into a genetic risk score (GRS) or “genetic instrument” that acts as a proxy for the specified trait during MR.

We conducted MR analysis by using data from the largest population-based studies in the United Kingdom (UK Biobank^2^) and Norway (Nord-Trøndelag Health Study, Norway^3^ [HUNT, 2006-08]) along with the largest published GWASs for BMI^4^^,^^5^ and AD^6^ to date, representing a total of 742,611 individuals (see Table E5 in this article's Online Repository at www.jacionline.org). One-sample MR was performed in the UK Biobank and HUNT data sets with the individuals’ BMI SNPs, measured BMI, and AD status. Two-sample MR using published GWAS data^4^, ^5^, ^6^ was performed and meta-analyzed with the 1-sample estimate to obtain an overall causal estimate. Similarly, reverse MR was conducted to investigate the effect of AD genetic risk on BMI. Methodologic details and sensitivity analysis are described in the Online Repository, including Fig E5.

The BMI GRS was strongly associated with BMI in both the UK Biobank and HUNT data sets (see Figs E6 and E7 in this article’s Online Repository at www.jacionline.org), supporting its use as a genetic instrument. Potential confounders of the GRS-BMI association were detected (see Figs E6-E9), but the magnitudes were minimal in comparison with the strength of association with BMI. Similarly, the AD GRS was a good predictor of AD in both the UK Biobank (OR = 1.26 [95% CI = 1.23-1.28]; F-statistic = 2036; *R*^*2*^ = 0.7%) and HUNT (OR = 1.15 [95% CI = 1.11-1.21]; F-statistic = 97; *R*^*2*^ = 0.4%) data sets, despite lacking an *FLG* null genotype (R501X/rs61816761), which is known to show strong association with AD.

Meta-analyzed 1- and 2-sample MRs show evidence of a small causal effect of higher BMI increasing the risk of AD (OR = 1.02 [95% CI = 1.00-1.04]; *P* = .03) (Fig 1). This represents an increase in AD risk by approximately 2% for each 1-kg/m^2^ increase in BMI, which is remarkably similar to the observational estimate (see Figs E2 and E3 in the Online Repository). Importantly, sensitivity analyses showed little evidence of pleiotropy (for details, see the Sensitivity Analysis section, Table E6, Fig E10, and Fig E11 in this article’s Online Repository at www.jacionline.org) or heterogeneity among the individual SNP effect estimates (UK Biobank Q = 101.07 [*P* = .32]; HUNT Q = 100.04 [*P* = .37]). Two-sample MR using the larger number of recently published BMI SNP estimates (941 SNPs)^4^ gave similar evidence of a causal effect on AD risk (OR = 1.08 [95% CI = 1.01-1.16]; *P* =.02).

**Fig 1:**
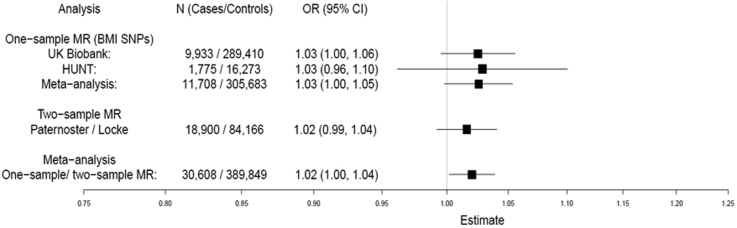
MR analysis of the causal effect of BMI on AD. Meta-analysis of 1-sample and 2-sample MR estimates using individual BMI SNPs as instruments. Estimates are given per 1-kg/m^2^ increase in BMI.

In the reverse direction, meta-analysis gave weak evidence of a very small causal effect (Fig 2): a 0.03-kg/m^2^ change in BMI per doubling odds of AD (95% CI = –0.02 to 0.08; *P* = .24). There was little evidence of pleiotropy but modest heterogeneity among the individual SNP effects (see Fig E12 and Table E7 in in this article’s Online Repository at www.jacionline.org). The difference in BMI between patients with AD and controls estimated in 1-sample MR (0.15 kg/m2, 95% CI -1.97 to 2.27) was small compared with observational estimates (see Tables E1 and E2), indicating that the association is mainly explained by the causal effect of BMI on AD.

**Fig 2:**
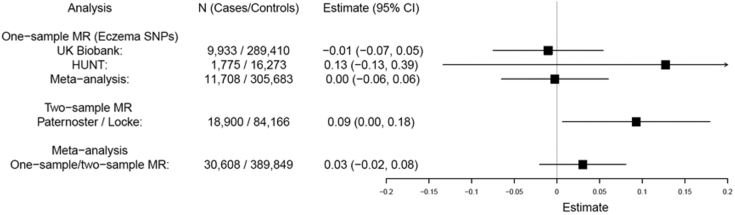
Reverse direction MR analysis: effect of AD genetic risk on BMI. Meta-analysis of 1-sample and 2-sample MR estimates using individual AD SNPs as instrumental variables. Estimates represent change in BMI (kg/m^2^) per doubling odds of AD.

The association of obesity with cardiometabolic disease and systemic inflammation is now well recognized, and clinical guidelines recommend screening patients with psoriasis for obesity. The presence of a *causal* effect and the *direction* of effect are both clinically relevant, to define a primary target (ie, obesity) for intervention. Our MR analysis shows evidence that higher BMI increases the risk of AD (ie, a 2% increase in disease risk for each 1-kg/m^2^ increase in BMI). Conversely, there was no strong evidence of a causal effect of AD genetic risk on BMI; the estimate of 0.03 kg/m^2^ suggests that genetic risk for AD has little meaningful influence on an individual’s BMI. These findings may be compared with the causal effect of BMI on psoriasis and lack of effect of psoriasis genetic risk on BMI.^1^ The effect of BMI on AD is more modest than the effect size observed in psoriasis, but the high prevalence of obesity (in more than one-third of US adults) and AD (in ≤10% of adults) demonstrate the potential importance of this causal effect on a population scale.

The molecular mechanisms by which obesity contributes to skin inflammation remain unclear. Excess adipose tissue secretes proinflammatory cytokines and hormones,^7^ and atopic inflammation may be promoted by disruption of the epidermal barrier in obese individuals.^8^ Changes in the adipocytes and lymphatic vessels may also contribute to obesity-related skin inflammation.^8^ Research to define mechanisms underlying the causal relationship demonstrated by MR may identify novel therapeutic targets.

Our MR analyses have various strengths as well as weaknesses. The large sample size is powerful, and the genetic instruments are strong. The 2-sample analysis included an overlap of data sources (from the HUNT study), which has the potential to bias the causal estimate, but this bias would be in the direction of the null. There is the possibility of misclassification of AD, and because AD often shows remission in childhood, this phenotype may be particularly susceptible to recall bias in adult studies; however, this would likely drive any estimate toward the null. It is also important to note that the MR methodology applied here does not define temporal relationship. Genetic risk has a lifetime effect and therefore predates disease onset, but a causal effect determined by MR does not rely on obesity occurring before the onset of AD. However, when we attempted to mitigate this issue by repeating the MR analyses using SNPs that are strongly associated with childhood BMI,^9^ a causal estimate with the same direction of effect was obtained (OR = 1.04; 95% CI = 1.01-1.07; *P =* .01). Nevertheless, replication of these analyses within pediatric cohorts with large sample sizes would be valuable future work.

In conclusion, we have found evidence of a small but potentially important causal effect of BMI on AD. Clinical trials have shown that interventions to promote weight loss can lead to improvement in psoriasis, but this approach has not been tested in AD. The results of our study provide support for the investigation of obesity management strategies and/or targeting of the adipocyte-keratinocyte cross-talk as therapeutic opportunities for AD. This may contribute to the prevention of AD, as well as to a reduction in the population prevalence of this chronic disease.

## Supplementary data

Online Repository
