# Supplementary material for: Assessment of a causal relationship between body mass index and atopic dermatitis
Source: J Allergy Clin Immunol. 2021 Jan;147(1):400–3. doi: 10.1016/j.jaci.2020.04.050 (PMC7794861; doi:10.1016/j.jaci.2020.04.050)
Supplement: Online Repository [file mmc1.pdf]

Assessment of a causal relationship between body mass index and atopic dermatitis

Budu-Aggrey *et al.*

Supplementary Methods and Results

Literature review

We searched for published studies that compared BMI, overweight or obesity rates between individuals with AD and controls. All studies identified in a PubMed search were considered for review. PubMed was searched on 18/11/2016 with the terms "(eczema OR atopic dermatitis AND (obesity OR overweight OR BMI)". We included studies with any operationalized definition of AD, since 'atopy' is often not clearly defined. We also included studies with cases meeting this definition of AD plus a control group without AD; that presented data for a BMI-related trait within the AD and control groups. Studies were excluded if they did not present data for both groups; if they matched individuals with AD and controls on BMI; or if cases and controls were both drawn from a disease sub-population. We did not exclude studies where participants may have had incidental comorbidities. We extracted the location of the study, the study name (if applicable), age of the study population, features of the control group (for example, if they were drawn from another dermatological population), the type of study, how AD was defined (if it was current, recent, or lifetime disease), the covariates used in the analysis, and the definition of overweight and/or obesity used by the study.

The definitions used for obesity and being overweight were based on international guidelines[1] or study-specific thresholds. Where stated, for the majority of studies BMI greater than 25 kg/m^2 was used to define being overweight, while BMI greater than 30 kg/m^2 was used to define obesity.

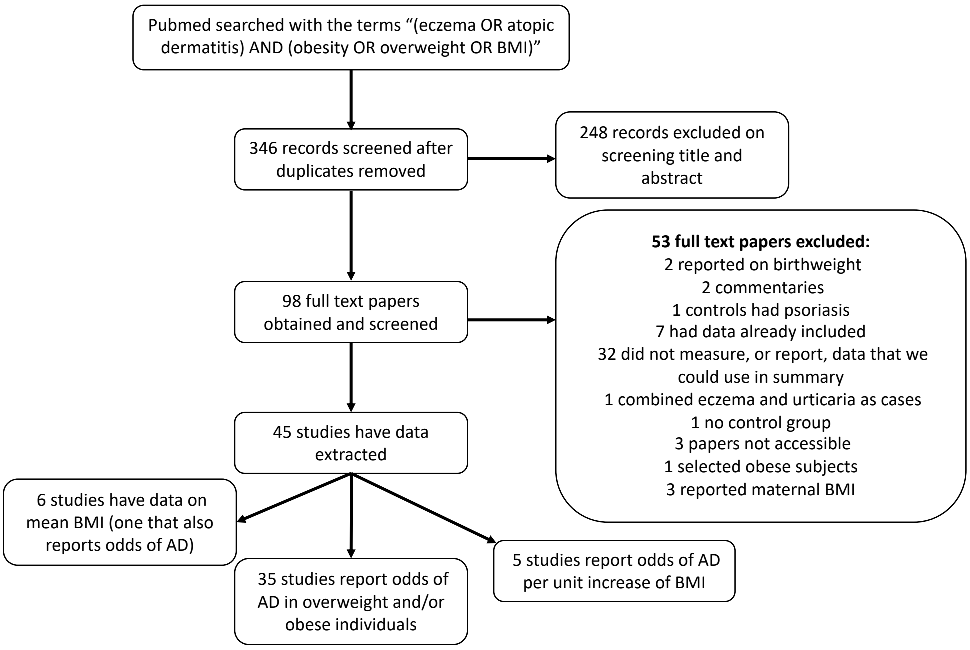

Figure E1. Flow diagram summarising literature review

Studies reporting the odds of AD in overweight and obese adults

Kilpeläinen M, Terho EO, Helenius H, et al. Body mass index and physical activity in relation to asthma and atopic diseases in young adults. Respir Med 2006;100:1518–25. doi:10.1016/J.RMED.2006.01.011

Silverberg JI, Silverberg NB, Lee-Wong M. Association between atopic dermatitis and obesity in adulthood. Br J Dermatol 2012;166:498–504. doi:10.1111/j.1365-2133.2011.10694.x

Gibeon D, Batuwita K, Osmond M, et al. Obesity-Associated Severe Asthma Represents a Distinct Clinical Phenotype: Analysis of the British Thoracic Society Difficult Asthma Registry Patient Cohort According to BMI. Chest 2013;143:406–14. doi:10.1378/CHEST.12-0872

Luo X, Xiang J, Dong X, et al. Association between obesity and atopic disorders in Chinese adults: an individually matched case-control study. BMC Public Health 2013;13:12. doi:10.1186/1471-2458-13-12

Sybilski AJ, Raciborski F, Lipiec A, et al. Obesity – a risk factor for asthma, but not for atopic dermatitis, allergic rhinitis and sensitization. Public Health Nutr 2015;18:530–6. doi:10.1017/S1368980014000676

Silverberg JI, Greenland P. Eczema and cardiovascular risk factors in 2 US adult population studies. J Allergy Clin Immunol 2015;135:721–728.e6. doi:10.1016/j.jaci.2014.11.023

Radtke MA, Schäfer I, Glaeske G, et al. Prevalence and comorbidities in adults with psoriasis compared to atopic eczema. J Eur Acad Dermatology Venereol 2017;31:151–7. doi:10.1111/jdv.13813

Rönmark EP, Ekerljung L, Mincheva R, et al. Different risk factor patterns for adult asthma, rhinitis and eczema: results from West Sweden Asthma Study. Clin Transl Allergy 2016;6:28. doi:10.1186/s13601-016-0112-0

Studies of AD in overweight and obese paediatric cases

von Kries R, Hermann M, Grunert VP, et al. Is obesity a risk factor for childhood asthma? Allergy 2001;56:318–22. doi:10.1034/j.1398-9995.2001.00727.x

Eneli IU, Karmaus WK, Davis S, et al. Airway hyperresponsiveness and body mass index: The child health and environment cohort study in Hesse, Germany. Pediatr Pulmonol 2006;41:530–7. doi:10.1002/ppul.20391

Mai X-M, Almqvist C, Nilsson L, et al. Birth anthropometric measures, body mass index and allergic diseases in a birth cohort study (BAMSE). Arch Dis Child 2007;92:881–6. doi:10.1136/adc.2006.110692

Kusunoki T, Morimoto T, Nishikomori R, et al. Obesity and the prevalence of allergic diseases in schoolchildren. Pediatr Allergy Immunol 2008;19:527–34. doi:10.1111/j.1399-3038.2007.00686.x

Leung TF, Kong APS, Chan IHS, et al. Association between obesity and atopy in Chinese schoolchildren. Int Arch Allergy Immunol 2009;149:133–40. doi:10.1159/000189196

Kajbaf TZ, Asar S, Alipoor MR. Relationship between obesity and asthma symptoms among children in Ahvaz, Iran: a cross sectional study. Ital J Pediatr 2011;37:1. doi:10.1186/1824-7288-37-1

Silverberg JI, Kleiman E, Lev-Tov H, et al. Association between obesity and atopic dermatitis in childhood: A case-control study. J Allergy Clin Immunol 2011;127:1180–1186.e1. doi:10.1016/J.JACI.2011.01.063

Tanaka K, Miyake Y, Arakawa M, et al. U-Shaped Association between Body Mass Index and the Prevalence of Wheeze and Asthma, but not Eczema or Rhinoconjunctivitis: The Ryukyus Child Health Study. J Asthma 2011;48:804–10. doi:10.3109/02770903.2011.611956

Yao T-C, Ou L-S, Yeh K-W, et al. Associations of Age, Gender, and BMI with Prevalence of Allergic Diseases in Children: PATCH Study. J Asthma 2011;48:503–10. doi:10.3109/02770903.2011.576743

Yoo S, Kim H-B, Lee S-Y, et al. Association between obesity and the prevalence of allergic diseases, atopy, and bronchial hyperresponsiveness in Korean adolescents. Int Arch Allergy Immunol 2011;154:42–8. doi:10.1159/000319207

Sidoroff V, Hyvärinen MK, Piippo-Savolainen E, et al. Overweight does not increase asthma risk but may decrease allergy risk at school age after infantile bronchiolitis. Acta Paediatr 2012;101:43–7. doi:10.1111/j.1651-2227.2011.02439.x

James S, Pezic A, Ponsonby A-L, et al. Obesity and asthma at school entry: Co-morbidities and temporal trends. J Paediatr Child Health 2013;49:E273–80. doi:10.1111/jpc.12160

Mitchell EA, Beasley R, Björkstén B, et al. The association between BMI, vigorous physical activity and television viewing and the risk of symptoms of asthma, rhinoconjunctivitis and eczema in children and adolescents: ISAAC Phase Three. Clin Exp Allergy 2013;43:73–84. doi:10.1111/cea.12024

Saadeh D, Salameh P, Caillaud D, et al. High body mass index and allergies in schoolchildren: the French six cities study. BMJ Open Respir Res 2014;1:e000054. doi:10.1136/bmjresp-2014-000054

Silverberg JI, Simpson EL. Association Between Obesity and Eczema Prevalence, Severity and Poorer Health in Us Adolescents. Dermatitis 2014;25:172–81. doi:10.1097/der.0000000000000047

Song N, Mohammed S, Zhang J, et al. Prevalence, severity and risk factors of asthma, rhinitis and eczema in a large group of Chinese schoolchildren. J Asthma 2014;51:232–42. doi:10.3109/02770903.2013.867973

Weinmayr G, Forastiere F, Büchele G, et al. Overweight/Obesity and Respiratory and Allergic Disease in Children: International Study of Asthma and Allergies in Childhood (ISAAC) Phase Two. PLoS One 2014;9:e113996. doi:10.1371/journal.pone.0113996

Augustin M, Radtke MA, Glaeske G, et al. Epidemiology and Comorbidity in Children with Psoriasis and Atopic Eczema. Dermatology 2015;231:35–40. doi:10.1159/000381913

Lee G, Ham OK. Factors Affecting Underweight and Obesity Among Elementary School Children in South Korea. Asian Nurs Res (Korean Soc Nurs Sci) 2015;9:298–304. doi:10.1016/J.ANR.2015.07.004

Lin M-H, Hsieh C-J, Caffrey JL, et al. Fetal Growth, Obesity, and Atopic Disorders in Adolescence: a Retrospective Birth Cohort Study. Paediatr Perinat Epidemiol 2015;29:472–9. doi:10.1111/ppe.12215

Silverberg JI. Association between adult atopic dermatitis, cardiovascular disease, and increased heart attacks in three population-based studies. Allergy 2015;70:1300–8. doi:10.1111/all.12685

Silverberg JI, Becker L, Kwasny M, et al. Central Obesity and High Blood Pressure in Pediatric Patients With Atopic Dermatitis. JAMA Dermatology 2015;151:144. doi:10.1001/jamadermatol.2014.3059

Sybilski AJ, Raciborski F, Lipiec A, et al. Obesity – a risk factor for asthma, but not for atopic dermatitis, allergic rhinitis and sensitization. Public Health Nutr 2015;18:530–6. doi:10.1017/S1368980014000676

Lei Y, Yang H, Zhen L. Obesity is a risk factor for allergic rhinitis in children of Wuhan (China). Asia Pac Allergy 2016;6:101. doi:10.5415/apallergy.2016.6.2.101

Six studies reported the mean difference in BMI between AD cases and controls (**Table E1**). These studies showed mixed results, but the majority (4 out of 6) reported mean BMI to be lower in AD cases compared to controls. Kusunoki *et al.* reported the odds of AD in children to be 1.02 per 1 kg/m^2 increase in BMI,[2] and the same direction of effect was observed in overweight and obese children (Fig 2). The remaining 5 studies reported the odds of AD with higher BMI (**Table E2**), where the majority (4 out of 5) estimates gave evidence of increased AD risk.

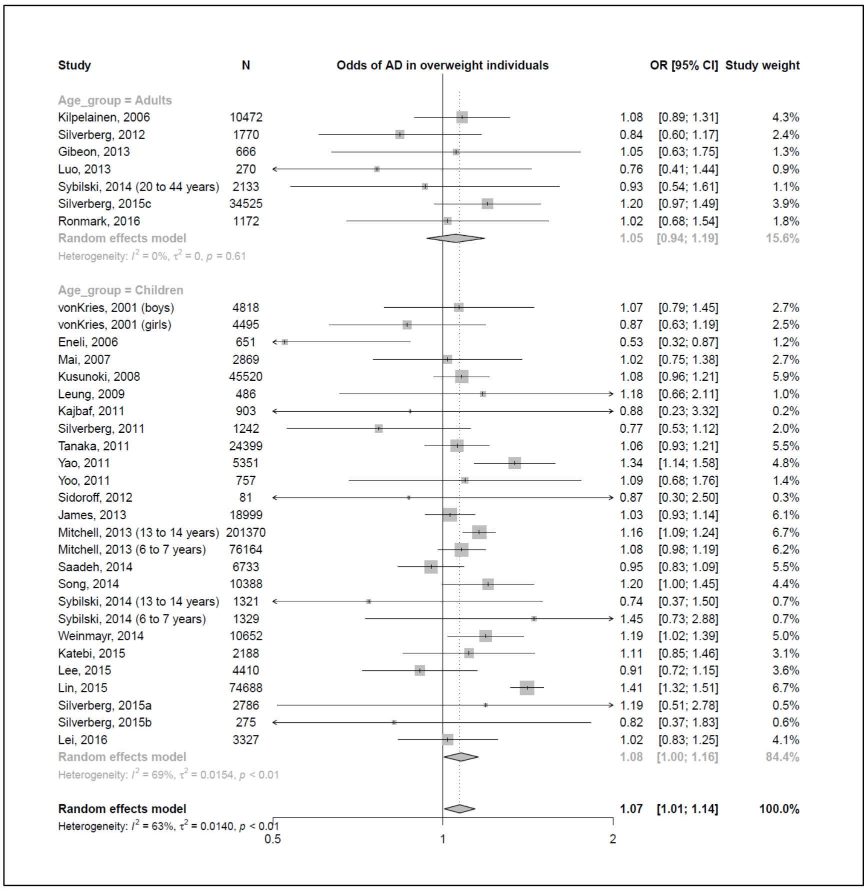

Figure E2. Observational association between being overweight and having AD: meta-analysis of the odds of AD in overweight individuals. CI, confidence interval; OR, odds ratios; top forest plot shows studies of adults, lower plot shows studies of children, final estimate is meta-analysis of both sets of data.

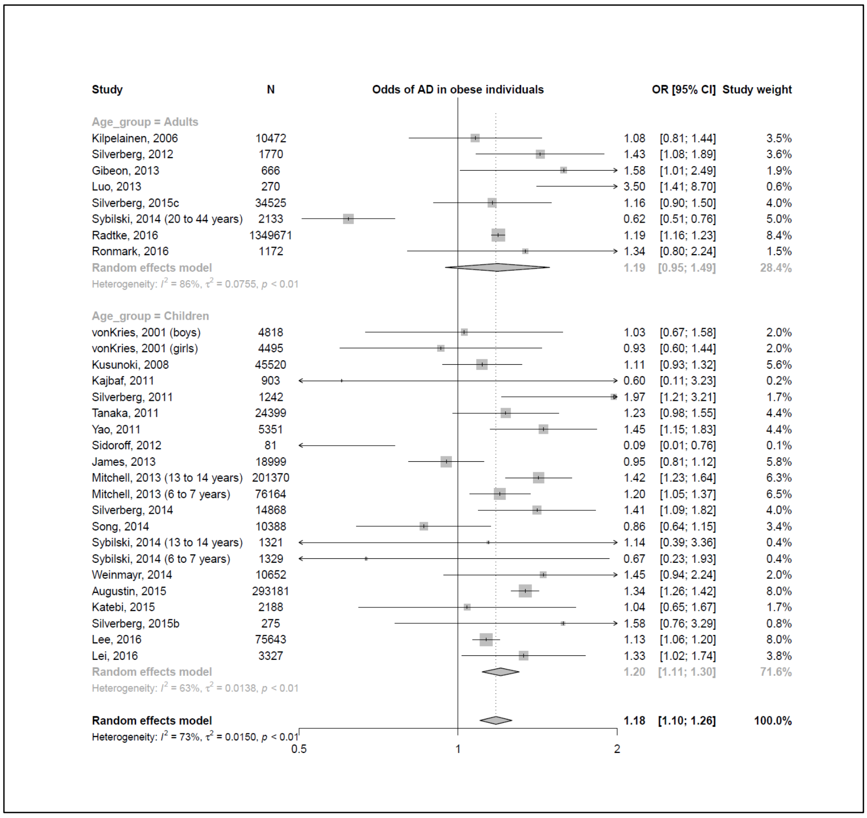

Figure E3. Observational association between obesity and AD: meta-analysis of the odds of AD in obese individuals. CI, confidence interval; OR, odds ratio; top forest plot shows studies of adults, lower plot shows studies of children, final estimate is meta-analysis of both sets of data.

Study	Age group	Mean difference (95% CI)
Ellison (Males), 2006	Children	0.44*
Ellison (Females), 2006	Children	-0.18*
Kusunoki, 2008	Children	-0.10 (-2.47, 2.27)
Machura, 2013	Children	-0.66 (-4.89, 3.57)
Kim, 2015	Adults	-0.06 (-3.89, 3.77)
Lee (Males), 2016	Adults	-0.20 (-6.66, 6.26)
Lee (Females), 2016	Adults	0.60 (-4.67, 5.87)
D'Auria, 2017	Children	-0.05*+

Table E1. Reviewed studies reporting the difference in BMI (kg/m^2) between AD cases and controls. CI, confidence interval; *Unable to estimate 95% confidence interval; +Mean difference in BMI z-score.

Study	Age group	OR (95% CI)
Lee, 2001	Children	1.29 (1.15, 1.44)
Irei, 2005	Children	1.02 (0.70, 1.47)
Purvis, 2005	Children (BMI >18 kg/m^2)	0.28 (0.07, 1.16)
Murray, 2010	Children	1.79* (1.03, 3.13)
Byberg, 2016	Children	1.32** (1.06, 1.65)

Table E2. Reviewed studies reporting the odds of AD per unit increase in BMI (kg/m^2) CI, confidence interval; *per unit increase in BMI z-score; **per unit increase in BMI standard deviation score

References

Ellison JA *et al*. Br J Dermatol 2006;155:532–8.
Kusunoki T *et al.* Pediatr Allergy Immunol. 2008;19:527–34.
Machura E *et al*. Mediators Inflamm. 2013:760691.
Kim S *et al.* Korean J Fam Med. 2015; 36:261.
Lee JH *et al.* Allergy Asthma Immunol Res. 2016;8:107–14.
D'Auria *et al*. Immunol let. 2017; 181:31-35.
Lee S Il *et al.*. J Korean Med Sci. 2001;16:155.
Irei A V *et al.* Eur J Clin Nutr. 2005;59:571–7.
Purvis DJ *et al.* Br J Dermatol. 2005;152:742–9.
Murray CS *et al.* Clin Exp Allergy. 2011;41:78–85.
Byberg KK *et al.* Clin Transl Allergy. 2016;6:33.

Meta-analysis of published studies

All data pertaining to BMI and AD were extracted and a meta-analysis was performed for the definition with the most available data, this being the odds of AD in overweight and/or obese individuals. This analysis represents an update of the 2015 review by Zhang *et al*.[3] The meta-analysis was conducted separately for children and adults, as well as combined. A random effects model was used due to the inclusion of heterogeneous populations and study designs being meta-analysed.

The overall OR for AD in overweight individuals was 1.05 (95% CI 0.94 to 1.19) in adults (n=51,008) and 1.08 (95% CI 1.00 to 1.16) in children (n=506,202) (**Figure E2**). For obese individuals, the OR for having AD was 1.19 (95% CI 0.95 to 1.49) in adults (n=1,400,679) and 1.20 (95% CI 1.11 to 1.30) in children (n=796,514) (**Figure E3**). Where stated, children were defined as those under 18 years of age, although one paediatric study included individuals aged up to 19 years.[4] Adults were defined as those aged 18 years and above in studies where this had been described.

Observational analysis using UK Biobank and HUNT datatsets

Clinical outcomes

The BMI of UK Biobank participants was calculated from standing height and weight measurements that were taken while visiting an assessment centre. Individuals were defined as having atopic dermatitis (AD) based on their response during a verbal interview with a trained member of staff at the assessment centre. Participants were asked to tell the interviewer which serious illnesses or disabilities they had been diagnosed with by a doctor and were defined as AD cases if this disease was mentioned. Disease information was also obtained from the Hospital Episode Statistics (HES) data extract service where health-related outcomes had been defined by International Classification of Diseases (ICD)-10 codes (**Table E3**). Additionally, if any had answered "yes" to "Has a doctor ever told you that you have hay fever, allergic rhinitis or eczema", then these individuals were excluded from the AD controls.

Phenotype	ICD10 codes
Atopic dermatitis	L20
Besnier's prurigo	L20-L200
Other atopic dermatitis	L20-L208
Atopic dermatitis, unspecified	L20-L209

Table E3. International Classification of Diseases (ICD)-10 codes used to obtain disease information for AD (eczema; atopic eczema; atopic dermatitis) from the UK Biobank resource

Within HUNT, participants' height and weight were measured and used to calculate BMI (kg/m^2). Participants were defined as AD cases based on their response to a general questionnaire sent to all HUNT participants. AD cases responded affirmatively to both "Have you had or do you have any of the following diseases: Eczema on hands" and "Did you have eczema when you were a child? (also called atopic eczema)". In addition, cases were obtained from the Nord-Trøndelag Health Trust which includes the two hospitals (Levanger and Namsos) in the study area, and the General Practitioner records. Disease classifications are shown in **Table E4**.

Table E4. International Classification of Diseases (ICD) and International Classification of Primary Care-2 (ICPC-2) codes used to obtain disease information for AD (eczema; atopic eczema; atopic dermatitis) from the HUNT study

Phenotype	ICD10	ICD9	ICPC-2
Atopic dermatitis	L20		
Other and unspecified dermatitis	L30		
Atopic dermatitis and related conditions		691	
Atopic dermatitis/eczema			S87

Confounder variables

Within UK Biobank, confounders that were considered in the current study were age, sex, smoking status, alcohol intake and educational attainment. The age and sex of participants were baseline characteristics determined at recruitment. The information on age was coded and analysed as a continuous variable, while sex was analysed as a binary variable. Smoking status, alcohol intake and educational attainment were defined by responses to a touchscreen questionnaire. The smoking status of participants was summarised as being a current or previous smoker, or never smoked, where this information was coded into a categorical variable. Alcohol intake frequency was determined by asking participants "about how often do you drink alcohol?", where options included "Daily or almost daily", "Three or four times a week", "One to three times a month", "Special occasions only" and "Never". This information was categorised for daily, weekly and monthly alcohol intake. Educational attainment was also defined by asking "which of the following qualifications do you have?", where participants could select more than one option including "College or University degree", "A levels/AS levels or equivalent", "O levels/GCSEs or equivalent", "CESs or equivalent", "NVQ or HND or HNC or equivalent", "Other professional qualifications eg: nursing, teaching", or "None of the above". Participant responses were coded into categorical variables for degree holders, those who had completed advanced level studies (A-level) or had obtained their general certificate of secondary education (GCSE). Within HUNT, confounders considered in the current study were age, sex, smoking status and alcohol intake. Information on educational attainment was not available in the third survey of the HUNT study. The age and sex of participants were determined at the time of participation. The information on age was coded and analysed as a continuous variable, while sex was analysed as a binary variable. Smoking status and alcohol intake were defined by the participants' response to a questionnaire. Smoking status was defined as being never, former, occasional, or current smoker. Alcohol intake frequency was determined by asking participants "about how often in the last 12 months did you drink alcohol?", where options included "4-7 times a week", "2-3 times a week", "about once a month", "a few times a year", "not at all last year" and "never drunk alcohol".

Meta-analysis

Within the UK Biobank and HUNT datasets, logistic regression models were used to estimate the observational association between BMI and AD. This analysis was performed for all individuals, as well as for overweight (25 kg/m2<BMI<30 kg/m2) and obese (BMI>30kg/m2) individuals alone. Analyses were adjusted for age, sex, smoking status, alcohol intake and educational attainment (where information on education was available in UK Biobank only). The estimates for each dataset were meta-analysed assuming a random effects model to account for heterogeneity.

Results

There was very little evidence of an association between BMI and AD in UK Biobank, but some evidence within the HUNT dataset (**Figure E4**). Upon meta-analysis, the OR of AD per 1 kg/m^2 higher BMI was 1.01 (95% CI 1.00 to 1.01; P=0.26). Among overweight individuals (BMI = 25 to 30 kg/m^2), the OR of AD per 1 kg/m^2 higher BMI was 1.02 (95% CI 1.00 to 1.04; P=0.07; 4,820 cases; 130,776 controls). A similar estimate was found among obese individuals (BMI greater than 30 kg/m^2) but with stronger evidence of an association (OR=1.02; 95% CI 1.01 to 1.03; P=3.3x10^{-4}; 2,741 cases and 73,907 controls).

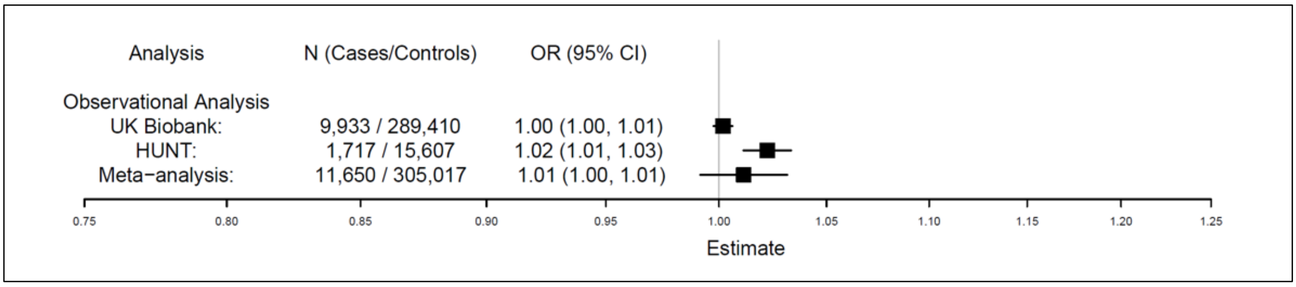

Figure E4. Observational association between BMI upon AD in two population-based studies. Association analysis and meta-analysis of observational data from the UK Biobank and HUNT study, Norway. Observational analysis was restricted to individuals with complete information on potential confounders. Estimates are given per 1kg/m^2 increase in BMI. CI, confidence interval.

Analysis of causal relationships

Study populations and phenotypes

Data were available, with written informed consent, for a total of 317,391 participants including 9,933 AD cases from the UK Biobank[5] aged 40-69 years and 1,775 AD cases from the third survey of the Nord-Trøndelag Health Study, Norway[6] (HUNT, 2006-08) aged 20 years and over (**Table E5**). AD in the UK Biobank was defined by patient-report of doctor diagnosis or hospital statistics using ICD-10 codes; in the HUNT study AD was defined by self-report and/or hospital statistics using ICD-9 and ICD-10 codes and/or diagnosed by general practitioners using ICPC-2 codes. Healthy controls were individuals from the same population-based studies, but without reported AD. All individuals included in this analysis were of white European ethnicity. The BMI (kg/m^2) of UK Biobank and HUNT participants was calculated from height and weight measurements. UK Biobank is approved by the National Health Service National Research Ethics Service (ref 11/NW/0382; UK Biobank application number 10074); the HUNT Study was approved by the Regional Committee for Medical and Health Research Ethics (REC Central), which also gave specific approval for this study (2015/2003). Summary level data were available for 425,220 individuals of European ancestry from published GWAS studies for BMI[7] (n=322,154) and AD[8] (n=103,066) (**Table E5**).

Dataset	Sample size	AD cases/controls (prevalence %)	Females (%)	Mean [SD] age (years)	Mean [SD] BMI (kg/m^2)
**UK Biobank**	299,343	9,933 / 289,410 (3.3)	158,103 (52.8)	57 [8.0]	27.4 [4.8]
**HUNT**	18,048	1,775/ 16,273 (9.9)	9,940 (55.1)	53.7 [15.2]	27.2 [4.4]
**BMI GWAS**[17]	322,154	-	-	-	27.1 [4.6]
**AD GWAS**[19]	103,066	18,900 / 84,166 (18.3)	-	-	-

Table E5. Descriptive statistics of datasets used in the study. HUNT, Nord-Trøndelag Health Study; SD, standard deviation.

Genotyping

Genotyping of UK Biobank participants was performed with one of two arrays (Applied Biosystems™ UK BiLEVE Axiom™ Array (Affymetrix) and Applied Biosystems™ UK Biobank Axiom™ Array). Sample quality control (QC) measures included removing individuals who were duplicated and highly related (third degree or closer), had sex mismatches, as well as those identified to be outliers of heterozygosity and of non-European descent. Further details of the QC measures applied, and imputation performed have been described previously.[9-11]

Genotyping of the HUNT participants was performed with one of three different Illumina HumanCoreExome arrays (HumanCoreExome12 v1.0, HumanCoreExome12 v1.1 and UM HUNT Biobank v1.0). The genotypes from different arrays had QC performed separately and were reduced to a common set of variants across all arrays. Sample QC measures were similar to those applied to the UK Biobank. Related individuals were excluded from the analysis (n=30,256). Details of the genotyping, QC and imputation are described elsewhere.[12]

Genetic instruments

The genetic instrument for BMI comprised the 97 BMI-associated SNPs reported by the GIANT consortium (a meta-analysis of 125 GWAS studies with 339,224 individuals).[7] We note that this study included 1,334 individuals from the HUNT dataset in their analysis. The 97 SNPs were extracted from both UK Biobank and HUNT datasets and combined to create a separate standardised genetic risk score (GRS) for each dataset using the --score command in PLINK (version 1.9). The dosage of the effect allele for each SNP was weighted by the effect estimates reported for the European sex-combined analysis (n= 322,154) by Locke *et al*,[7] summed across all variants and divided by the total number of variants. The scores were standardized to have a mean of 0 and standard deviation of 1. One BMI-associated SNP, rs12016871, was not present within the UK Biobank and HUNT datasets, therefore rs9581854 was used as a highly correlated proxy (r^2 = 1.0). Of the 97 BMI SNPs, 22 are strongly associated with BMI in childhood[13] and since AD is a predominantly paediatric disease we used these SNPs as genetic instruments for BMI in childhood. The associations of BMI genetic instruments with BMI in UK Biobank and HUNT and associations of BMI genetic instruments with atopic dermatitis (AD) in UK Biobank and HUNT (using BMI variants reported by Locke *et al*. 2015) are available in GitHub (https://github.com/abudu-aggrey/Eczema_BMI_MR).

In addition, the BMI-associated SNPs most recently reported by Yengo *et al*[14] were used as an updated genetic instrument for BMI, to include novel BMI variants which may strengthen the genetic instrument. However, this study included individuals from the UK Biobank and was therefore not our preferred genetic instrument for the main analysis.

For the AD genetic instrument, 24 SNPs that had been reported within white European populations in the most recent AD GWAS (meta-analysis of 21,399 cases and 95,464 controls)[8] were used. This GWAS did not contain individuals from UK Biobank or HUNT (**Table E5**). The associated SNPs were extracted from UK Biobank and HUNT datasets and combined to create a standardized GRS for each dataset, weighted by the published effect estimates reported for European individuals (18,900 cases; 84,166 controls).[8] Within the HUNT dataset, the AD-associated SNP rs12153855 was not present and no suitable proxy (r^2 > 0.8) was found, therefore 23 SNPs were used to create the genetic instrument in the HUNT analysis.

The BMI GRS was strongly associated with BMI in both UK Biobank (Beta=0.64; 95% CI 0.63 to 0.66, F-statistic=7091, R^2=1.8%) and HUNT (Beta=0.66; 95% CI 0.60 to 0.72, F-statistic=422, R^2=2.3%). Likewise, a GRS of the childhood BMI SNPs was also strongly associated with BMI in UK Biobank (Beta=0.48; 95% CI 0.47 to 0.50, F-statistic=185, R^2=2.6%). The AD GRS was found to be a good predictor of AD in both UK Biobank (OR=1.26; 95% CI 1.23 to 1.28, F-statistic=2036, R^2=0.7%) and HUNT (OR=1.15; 95% CI 1.11 to 1.21, F-statistic=97, R^2=0.4%) datasets.

MR: investigation of the effect of BMI on AD

One-sample MR analysis was performed in the UK Biobank and HUNT datasets with the individuals' BMI SNPs, measured BMI and AD status (**Figure E5(a)**). The MR estimates from each SNP were meta-analysed assuming a random effects model, giving a single estimate for the analysis performed in each dataset. A random effects model was used to avoid over-precision of the causal estimate, and to allow for heterogeneity in the causal estimates being meta-analysed from the different genetic variants.

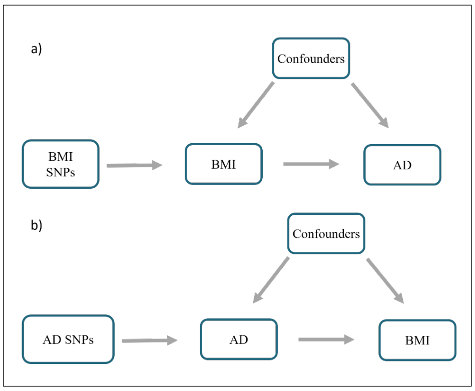

Figure E5. Schematic representation of MR analyses.

a) BMI SNPs were used as instrumental variables to investigate the causal effect of BMI upon AD.

b) AD SNPs were used as instrumental variables to investigate the causal effect of AD genetic risk of upon BMI. Arrows indicate MR assumptions where the instrumental variable is associated with the exposure, not associated with confounders, and only affects the outcome via the exposure. AD, atopic dermatitis; BMI, body mass index; SNP, single nucleotide polymorphism.

The MR analysis with the individual BMI SNPs was performed with the two-stage predictor substitution (TSPS) method.[15] The first stage involved regression of BMI upon individual BMI SNPs. The outcome (AD) was then regressed upon the fitted values from the first regression stage. As AD is a binary outcome, the first stage linear regression was restricted to control individuals, as recommended by Burgess *et al.*[16] Logistic regression was then performed in the second stage where the fitted values for the cases were predicted. The standard errors (SE) of these estimates were adjusted using the first term of the delta method expansion for the variance of a ratio, allowing for the uncertainty in the first regression stage to be considered.[16] Genetic principal components (as previously described[11, 12]) were included as covariates in the analysis to control for residual population structure. UK Biobank analysis also controlled for the platform used to genotype the samples. A similar protocol was followed in HUNT adjusting for the first four principal components and genotyping batch.

Two-sample MR analysis of published GWAS data was performed using the "TwoSampleMR" R package.[17] Estimates for the association between BMI and BMI SNPs in Europeans were taken from the GIANT BMI GWAS study published by Locke and colleagues.[7] Summary statistics from the most recent AD GWAS meta-analysis[8] were used to obtain estimates for the association of AD with the BMI SNPs in Europeans. The published BMI SNP estimates were based on an inverse normal transformation of BMI residuals on age and age-squared, as well as any necessary study-specific covariates. In unrelated individuals, residuals were calculated according to sex and case/control status and were sex-adjusted amongst related individuals. The causal estimates for the two-sample analysis were converted to raw BMI units (kg/m^2), assuming 4.6kg/m^2 to be the median BMI standard deviation.[7] The one-sample estimates obtained from UK Biobank and HUNT were meta-analysed assuming a fixed effect model. This was then meta-analysed with the two-sample estimate to obtain an overall causal estimate, assuming no between-method heterogeneity. This was performed using the genetic instrument of 97 BMI-associated SNPs, and separately for the instrument of 22 childhood BMI-associated SNPs. A separate two-sample MR analysis was performed in the same manner, using BMI SNP-BMI association estimates from the more recent BMI meta-analysis reported by Yengo and colleagues.[14]

Asessment of confounders

When investigating the association between the BMI GRS and potential confounders of BMI, some small associations with the confounders were seen (**Figures E6 and E7**). However, the magnitudes of these associations were minimal in comparison to the strength of association with BMI. The FTO variant alone also showed some effect on confounders, but it was much more strongly associated with measured BMI than potential confounders (**Figures E8 and E9**).

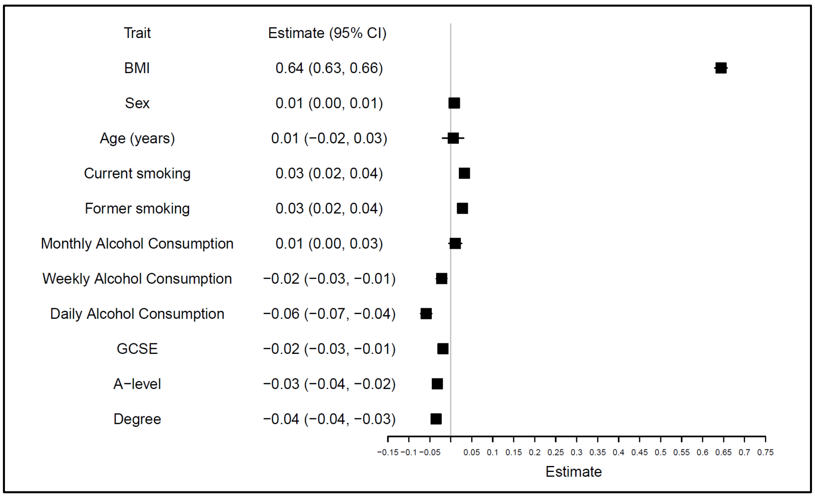

Figure E6. Association of BMI genetic risk score with BMI (kg/m^2) and potential confounders in UK Biobank. Estimates are given per 1 standard deviation increase in BMI GRS. A-level, Advanced level studies; CI, confidence interval; GCSE, General Certificate of Secondary Education; Monthly Alcohol Consumption was defined as frequency of “one to three times a month”. For “sex”, reference = Female”.

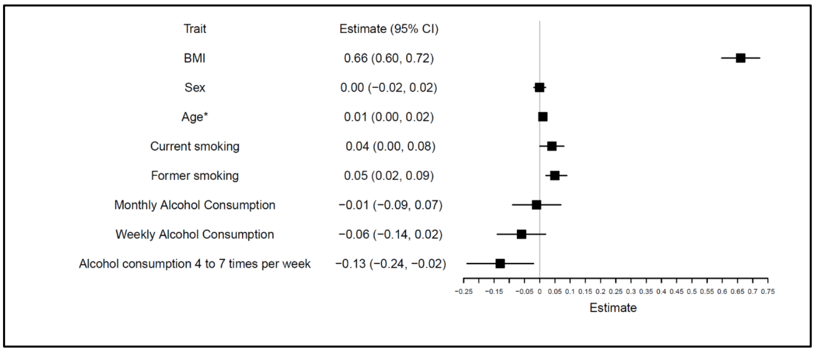

Figure E7. Association of BMI GRS with BMI (kg/m^2) and potential confounders in HUNT. Estimates are given per 1 standard deviation increase in BMI GRS. BMI, body mass index; CI, confidence interval.*age given per 10-year intervals. For “sex”, reference = Female”.

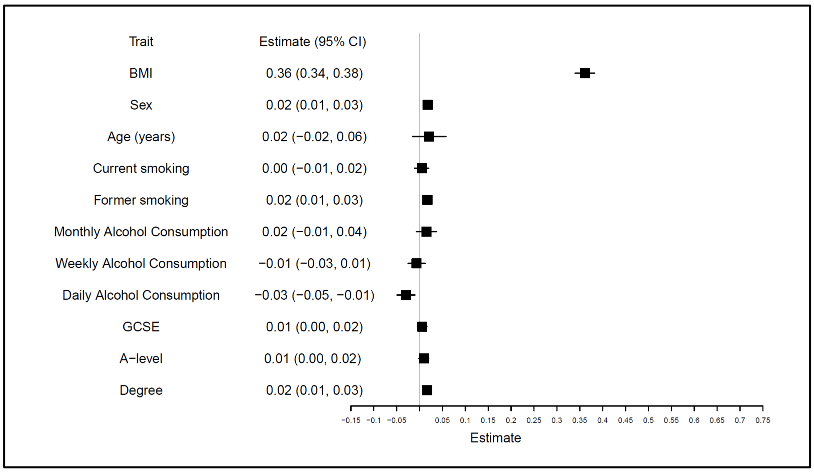

Figure E8. Association of FTO SNP (rs1558902) with BMI (kg/m^2) and potential confounders in UK Biobank. Estimates are given per 1 copy increase in effect allele (*A*). A-level, Advanced level studies; CI, confidence interval; GCSE, General Certificate of Secondary Education; Monthly Alcohol Consumption, defined as frequency of “one to three times a month”. For “sex”, reference = Female”.

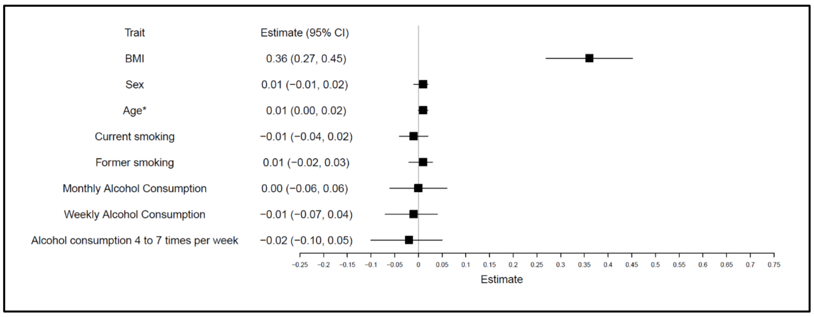

Figure E9. Association of FTO SNP (rs1558902) with BMI (kg/m^2) and potential confounders in HUNT. Estimates are given per 1 copy increase in effect allele (*A*). *age given per 10-year intervals. For “sex”, reference = Female”.

Sensitivity analysis

MR-Egger regression, weighted median analysis and the weighted mode-based estimate (MBE) were used to investigate potential horizontal pleiotropy. The strict definition of pleiotropy is when a SNP influences more than one trait.[18] In MR, vertical pleiotropy is assumed where the genetic instrument influences the exposure which in turn influences the outcome. However, SNPs that influence the exposure and the outcome through different pathways, known as horizonal pleiotropy, would violate the MR assumption that the instrumental variable has an effect on the outcome only via the exposure being investigated and could bias the causal estimate.[19] The weighted median method provides a valid causal estimate if at least 50% of the information in the MR analysis comes from valid instruments.[20]

Likewise, the weighted MBE also provides a valid causal estimate based on the assumption that the most frequent pleiotropy value is zero across the genetic instruments,[21] whilst the intercept from the MR-Egger regression analysis allows the size of any pleiotropic effect to be determined.[22] MR-Egger regression gives a valid causal estimate under the 'InSIDE' assumption, where each SNP-exposure effect is uncorrelated with the horizontal pleiotropic effect of the SNP.[22] We also used heterogeneity statistics to detect invalid instruments in MR that are due to the presence of pleiotropy.[23] As a proof-of-concept, one-sample MR analysis was performed using the *FTO* SNP alone (rs1558902) as a genetic instrument due to its strong association with BMI.[24, 25] The instrumental variables used in an MR analysis are assumed to be independent of confounders to avoid bias of the causal estimate. We therefore investigated the relationship between the BMI GRS, the *FTO* variant alone, and potential confounders of BMI by performing a simple regression of the confounder upon the BMI GRS and *FTO* variant.

Reverse MR: investigating the effect of AD genetic risk on BMI

In this reverse MR, we investigated the genetic liability of AD upon BMI (**Figure E5(b)**). One-sample MR analysis was performed separately in the UK Biobank and HUNT datasets using the two-stage least squares (TSLS) method with individual AD SNPs as genetic instruments. This analysis involves two regression stages where AD is first regressed upon the instrument (disease-associated SNPs), then the outcome (BMI) is regressed upon the fitted values from the first stage regression.[19] The final one-sample MR estimates from UK Biobank and HUNT were meta-analysed assuming a fixed effect model to give a single causal estimate (change in BMI per log odds of AD). To aid interpretation of the causal estimate, one-sample MR was also performed where genetic liability for AD was considered as a continuous variable, with values from "0" to "1" to give the difference in BMI between AD cases and controls with the "ivpack" R package.[26] Two-sample MR analysis was performed with the "TwoSampleMR" R package,[17] using summary results from GWAS studies for AD[8] and BMI.[7] The one- and two-sample MR estimates were meta-analysed using a fixed effect model to give an overall causal estimate. To aid interpretation, these estimates were multiplied by 0.693 to give the change in BMI per doubling odds of AD, as demonstrated by Gage *et al*.[27] Sensitivity analyses for the reverse MR were performed using MR-Egger regression, weighted median and weighted MBE analysis methods. A two-sample MR analysis was performed in the same manner, where AD SNP-BMI association estimates were extracted from the more recent BMI meta-analysis.[14]

Analysis software

All analyses were performed using R (www.r-project.org) unless otherwise stated. The code used to carry out these analyses is available on GitHub (https://github.com/abudu-aggrey/Eczema_BMI_MR).

Results of MR analyses

Causal effect of BMI upon AD

Similar causal estimates were found in UK Biobank (OR=1.03; 95% CI 1.00 to 1.06; *P*=0.10) and HUNT (OR=1.03; 95% CI 0.96 to 1.10; *P*=0.41). The estimate from the two-sample analysis with published GWAS data gave limited evidence of higher BMI increasing AD risk (OR=1.02; 95% CI 0.99 to 1.04; *P*=0.19).

Meta-analysis of the UK Biobank, HUNT and two-sample estimates showed evidence of a small causal effect, OR=1.02 (95% CI 1.00 to 1.04; *P*=0.03) (**Figure 1 in Letter**). This represents an increase in the odds of AD by ~2% for each 1 unit increase in BMI, or an increase in the risk of AD by approximately 11% for an increase in BMI of 5 units (OR per 5 units higher BMI = exp(Beta per 1 unit higher BMI * 5), for example, from 20 to 25kg/m^2. A similar causal estimate was found when restricting the BMI instrument to SNPs most strongly associated with childhood BMI (OR=1.04; 95% CI 1.01 to 1.07; *P*=0.01).

Sensitivity analyses

MR-Egger regression analysis showed little evidence of pleiotropy (UK Biobank intercept= 0.00; 95% CI -0.01 to 0.01; *P*=0.80, HUNT intercept=0.01; 95% CI -0.01 to 0.02; *P*=0.34) and the sensitivity analyses gave similar estimates (**Table E6, Figure E10**). There was also little evidence of heterogeneity among the individual effect estimates for each SNP in both datasets (UK Biobank Q=101.07, *P*=0.32; HUNT Q=100.04, *P*=0.37). MR analysis with the *FTO* SNP alone gave a slightly stronger estimate but with a wider confidence interval (OR=1.05; 95% CI 0.98 to 1.12; *P*=0.20) (**Figure E11**). Performing two-sample MR with the larger number of most recently published BMI SNP estimates (941 SNPs)[14] also gave evidence of a causal effect upon AD risk (OR=1.08; 95% CI 1.01 to 1.16; *P*=0.02).

Effect of AD genetic risk upon BMI

One-sample MR estimates in UK Biobank and HUNT gave little evidence that genetic risk of AD influences BMI (0.00kg/m^2 change in BMI per doubling odds of AD, 95% CI -0.06 to 0.06, *P*=0.94). Meta-analysis with the two-sample estimate also gave weak evidence of a very small causal effect (0.03 kg/m^2 change in BMI per doubling odds of AD, 95% CI -0.02 to 0.08, *P*=0.24). However, we note that the *FLG* loss-of-function variant (R501X/rs61816761) known to be strongly associated with AD[28] was not available in the two-sample analysis. There was little evidence of pleiotropy in the causal estimate for genetic risk of AD on BMI, and modest heterogeneity among the individual SNP effects (UK Biobank Q=66.51, *P*=4.15×10^{-6}; HUNT Q=24.10, *P*=0.34) (**Table E7, Figure E12**).

When performing two-sample MR with summary data for the most recently reported BMI SNPs,[14] the causal difference in BMI also gave very little evidence for BMI being influenced by genetic risk for AD, (0.02 kg/m^2 lower BMI in cases compared with controls, 95% CI -0.12 to 0.15, P=0.82). It should be noted, however, that summary statistics were not available for 5 of the 24 AD-associated SNPs in this analysis, including the *FLG* loss-of-function variant (R501X/rs61816761), thereby weakening the genetic instrument.

Table E6. Sensitivity analyses for the causal effect of BMI upon AD.

Dataset	TSPS OR (95% CI)	IVW OR (95% CI)	MR-Egger OR (95% CI)	Weighted Median OR (95% CI)	Weighted MBE OR (95% CI)
UK Biobank	1.03 (1.00, 1.06)	1.03 (1.00, 1.06)	1.03 (0.97, 1.10)	1.02 (0.97, 1.07)	1.02 (0.97, 1.08)
HUNT	1.03 (0.96, 1.10)	1.04 (0.97, 1.11)	0.99 (0.88, 1.11)	0.99 (0.89, 1.09)	0.99 (0.82, 1.20)

OR per 1 unit increase in BMI (kg/m^2); CI, confidence interval; IVW, inverse variance weighted analysis; MBE, mode-based estimate; OR, odds ratio; TSPS, two-stage predictor substitution.

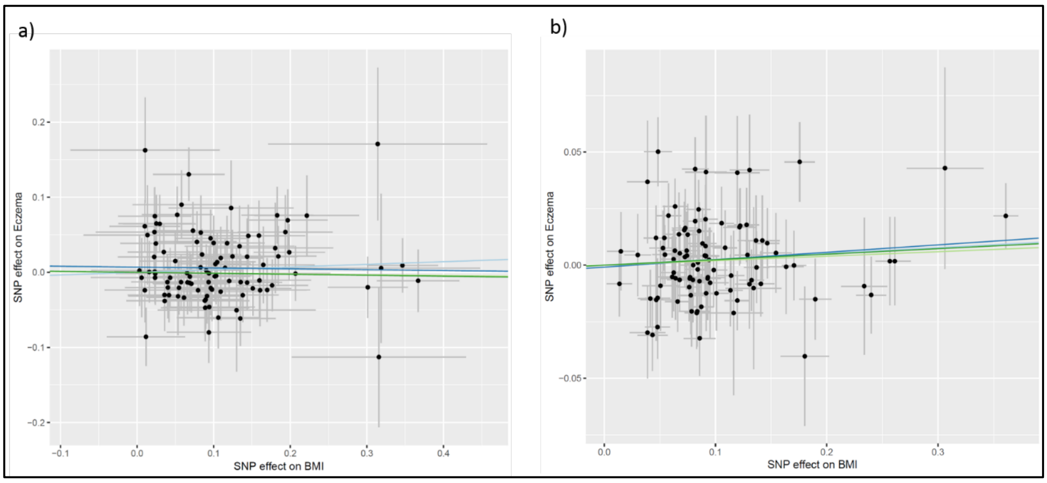

Figure E10. Associations of BMI SNPs with BMI and AD within a) UK Biobank b) HUNT. IVW, MR-Egger, weighted median and weighted mode estimates are indicated by the light blue, dark blue, light green, and dark green lines respectively.

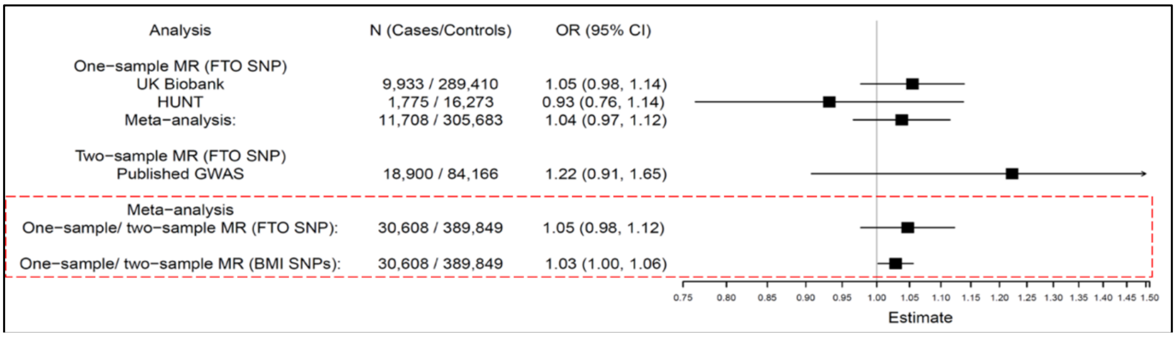

Figure E11. Effect of FTO variant, rs1558902 upon AD. Results from MR analysis using rs1558902 as an instrumental variable. These are compared with final estimates obtained when using all BMI SNPs as a genetic instrument. Estimates are given per 1 unit increase in BMI (kg/m^2).

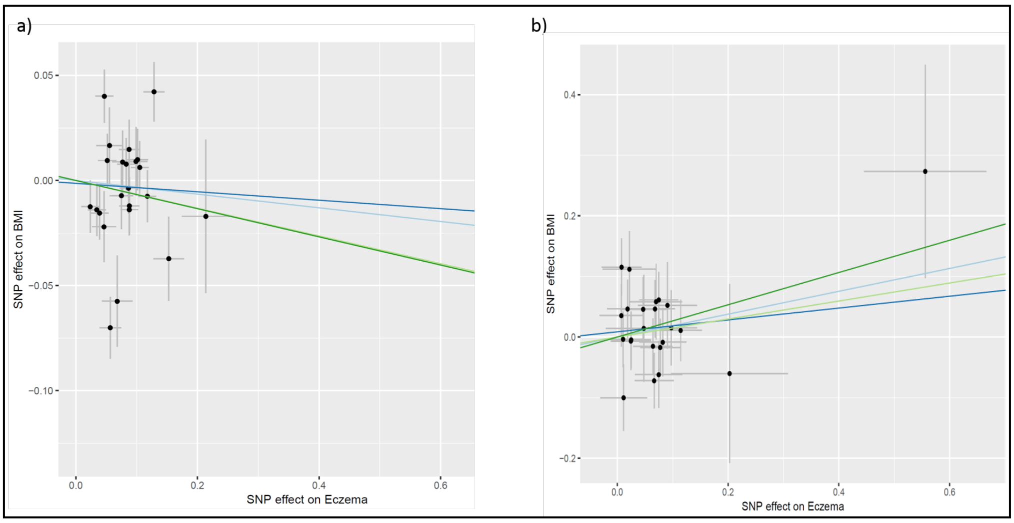

Figure E12. Association of AD SNPs with AD and BMI within (a) UK Biobank and (b) HUNT. IVW, MR-Egger, weighted median and weighted mode estimates are indicated by the light blue, dark blue, light green, and dark green lines respectively.

Table E7. Sensitivity analyses for the causal effect of AD genetic risk upon BMI.

Dataset	TSLS estimate* (95% CI)	IVW estimate** (95% CI)	MR-Egger estimate (95% CI)	Weighted Median estimate (95% CI)	Weighted MBE (95% CI)
UK Biobank	-0.01 (-0.07, 0.05)	-0.10 (-0.44, 0.23)	-0.06 (-0.71, 0.59)	-0.21 (-0.51, 0.09)	-0.21 (-0.56, 0.13)
HUNT	0.13 (-0.13, 0.39)	0.60 (-0.44, 1.64)	0.31 (-1.29, 1.91)	0.47 (-1.01, 1.96)	0.85 (-0.60, 2.30)

Change in BMI (kg/m^2) per doubling odds of atopic dermatitis; CI, confidence interval; IVW, inverse variance weighted analysis; MBE, mode-based estimate; TSLS, two-staged least squares.

References

1. Cole TJ, Bellizzi MC, Flegal KM, Dietz WH. Establishing a standard definition for child overweight and obesity worldwide: international survey. BMJ 2000; 320:1240-3.
2. Kusunoki T, Morimoto T, Nishikomori R, Heike T, Ito M, Hosoi S, et al. Obesity and the prevalence of allergic diseases in schoolchildren. Pediatr Allergy Immunol 2008; 19:527-34.
3. Zhang A, Silverberg JI. Association of atopic dermatitis with being overweight and obese: a systematic review and metaanalysis. J Am Acad Dermatol 2015; 72:606-16 e4.
4. Silverberg JI. Association between adult atopic dermatitis, cardiovascular disease, and increased heart attacks in three population-based studies. Allergy 2015; 70:1300-8.
5. Sudlow C, Gallacher J, Allen N, Beral V, Burton P, Danesh J, et al. UK biobank: an open access resource for identifying the causes of a wide range of complex diseases of middle and old age. PLoS Med 2015; 12:e1001779.
6. Krokstad S, Langhammer A, Hveem K, Holmen TL, Midthjell K, Stene TR, et al. Cohort Profile: the HUNT Study, Norway. Int J Epidemiol 2013; 42:968-77.
7. Locke AE, Kahali B, Berndt SI, Justice AE, Pers TH, Day FR, et al. Genetic studies of body mass index yield new insights for obesity biology. Nature 2015; 518:197-206.
8. Paternoster L, Standl M, Waage J, Baurecht H, Hotze M, Strachan DP, et al. Multi-ancestry genome-wide association study of 21,000 cases and 95,000 controls identifies new risk loci for atopic dermatitis. Nat Genet 2015; 47:1449-56.
9. Wain LV, Shrine N, Miller S, Jackson VE, Ntalla I, Soler Artigas M, et al. Novel insights into the genetics of smoking behaviour, lung function, and chronic obstructive pulmonary disease (UK BiLEVE): a genetic association study in UK Biobank. Lancet Respir Med 2015; 3:769-81.
10. Bycroft C, Freeman C, Petkova D, Band G, Elliott LT, Sharp K, et al. The UK Biobank resource with deep phenotyping and genomic data. Nature 2018; 562:203-9.
11. UK Biobank Genetic Data: MRC-IEU Quality Control. 2017.] Available from https://data.bris.ac.uk/datasets/3074krb6t2frj29yh2b03x3wxj/UK.
12. Nielsen JB, Thorolfsdottir RB, Fritsche LG, Zhou W, Skov MW, Graham SE, et al. Biobank-driven genomic discovery yields new insight into atrial fibrillation biology. Nat Genet 2018; 50:1234-9.
13. Felix JF, Bradfield JP, Monnereau C, van der Valk RJ, Stergiakouli E, Chesi A, et al. Genome-wide association analysis identifies three new susceptibility loci for childhood body mass index. Hum Mol Genet 2016; 25:389-403.
14. Yengo L, Sidorenko J, Kemper KE, Zheng Z, Wood AR, Weedon MN, et al. Meta-analysis of genome-wide association studies for height and body mass index in approximately 700000 individuals of European ancestry. Hum Mol Genet 2018; 27:3641-9.
15. Burgess S, Collaboration CCG. Identifying the odds ratio estimated by a two-stage instrumental variable analysis with a logistic regression model. Stat Med 2013; 32:4726-47.
16. Burgess S, Small DS, Thompson SG. A review of instrumental variable estimators for Mendelian randomization. Stat Methods Med Res 2017; 26:2333-55.
17. Hemani G, Zheng J, Elsworth B, Wade KH, Haberland V, Baird D, et al. The MR-Base platform supports systematic causal inference across the human phenome. Elife 2018; 7.
18. Hemani G, Bowden J, Davey Smith G. Evaluating the potential role of pleiotropy in Mendelian randomization studies. Hum Mol Genet 2018; 27:R195-R208.
19. Budu-Aggrey A, Paternoster L. Research Techniques Made Simple: Using Genetic Variants for Randomization. J Invest Dermatol 2019; 139:1416-21 e1.
20. Bowden J, Davey Smith G, Haycock PC, Burgess S. Consistent Estimation in Mendelian Randomization with Some Invalid Instruments Using a Weighted Median Estimator. Genet Epidemiol 2016; 40:304-14.
21. Hartwig FP, Davey Smith G, Bowden J. Robust inference in summary data Mendelian randomization via the zero modal pleiotropy assumption. Int J Epidemiol 2017; 46:1985-98.
22. Bowden J, Davey Smith G, Burgess S. Mendelian randomization with invalid instruments: effect estimation and bias detection through Egger regression. Int J Epidemiol 2015; 44:512-25.
23. Bowden J, Hemani G, Davey Smith G. Invited Commentary: Detecting Individual and Global Horizontal Pleiotropy in Mendelian Randomization-A Job for the Humble Heterogeneity Statistic? Am J Epidemiol 2018; 187:2681-5.
24. Speliotes EK, Willer CJ, Berndt SI, Monda KL, Thorleifsson G, Jackson AU, et al. Association analyses of 249,796 individuals reveal 18 new loci associated with body mass index. Nat Genet 2010; 42:937-48.
25. Loos RJ, Yeo GS. The bigger picture of FTO: the first GWAS-identified obesity gene. Nat Rev Endocrinol 2014; 10:51-61.
26. Baiocchi M, Cheng J, Small DS. Instrumental variable methods for causal inference. Stat Med 2014; 33:2297-340.
27. Gage SH, Jones HJ, Taylor AE, Burgess S, Zammit S, Munafo MR. Investigating causality in associations between smoking initiation and schizophrenia using Mendelian randomization. Sci Rep 2017; 7:40653.
28. Rodriguez E, Baurecht H, Herberich E, Wagenpfeil S, Brown SJ, Cordell HJ, et al. Meta-analysis of filaggrin polymorphisms in eczema and asthma: robust risk factors in atopic disease. J Allergy Clin Immunol 2009; 123:1361-70 e7.
